# Should the existing science of teams be applied to fluid teams? An exploration of fluid team effectiveness within the context of healthcare simulation

**DOI:** 10.3389/fpsyg.2024.1323469

**Published:** 2024-02-01

**Authors:** Rebecca Grossman, Brianna M. Billotti, Joseph J. Ha, Michael Cassara

**Affiliations:** ^1^Department of Psychology, Hofstra University, Hempstead, NY, United States; ^2^Northwell, New Hyde Park, NY, United States; ^3^Center for Learning and Innovation, New Hyde Park, NY, United States; ^4^Donald and Barbara Zucker School of Medicine at Hofstra/Northwell, Hempstead, NY, United States

**Keywords:** fluid teams, team effectiveness, healthcare teams, thematic analysis, critical incidents

## Abstract

**Introduction:**

Fluid teams have become increasingly prevalent and necessary for modern-day issues, yet they differ from more traditional teams, on which much of the current teams literature is based. For example, fluid teams are often comprised of members from different disciplines or organizational divisions who do not have a shared history or future, as they come together to perform a critical, time-sensitive task, and then disband. For these reasons, the mechanisms through which they function and perform may differ from those of more traditional teams, and research is needed to better understand these differences.

**Methods:**

To this end, this study utilized critical incident techniques and thematic analysis to examine fluid teams within healthcare, one of the primary contexts in which they are prevalent. Interdisciplinary faculty and students in the medical field who encounter fluid teams within simulation-based education were prompted to reflect on key factors that facilitate or hinder fluid team effectiveness.

**Results:**

Primary themes extracted pertained to the conditions fluid teams operate within (e.g., high-stress), the behaviors and emergent states that contribute to their success (e.g., communication), and the KSAO’s of value for members of fluid teams to possess (e.g., readiness). These themes were then compared to existing literature, yielding the identification of some similarities but also many important differences between fluid and traditional teams.

**Discussion:**

A series of practical recommendations for how to promote fluid team effectiveness is then presented.

## Introduction

As the building blocks of organizations around the globe today, we have come to know a lot about teams (Shuffler et al., 2018; Mathieu et al., 2019). Decades of science have accumulated, providing valuable insights on how teams function and the numerous factors that influence their effectiveness. However, teams can exist in many forms and are continually changing (Tannenbaum et al., 2012; Benishek and Lazzara, 2019). Thus, there are various team structures we know much less about in the literature, one of them being fluid teams. Fluid teams are those that are assembled in an *ad hoc* manner to perform critical, often time-sensitive tasks, and then disband upon completion of their mission (Bushe and Chu, 2011). They are typically composed of members with varying types and levels of expertise, from different organizational divisions, who may have little to no prior familiarity or experience working together. To address the complexity and ever-changing needs of the modern day, fluid teams have become increasingly common. They are the mechanism through which work is accomplished in numerous contexts, such as healthcare, aviation, disaster response, and even technology and other knowledge-based industries (Bushe and Chu, 2011).

However, fluid teams clearly differ from more traditional teams, on which much of the current literature is based. For example, traditional, intact teams are relatively stable over time, enabling members to develop familiarity and a shared history of working together (Bell et al., 2018). In addition, they do not necessarily operate under the high-stakes, time-sensitive conditions characteristic of fluid teams. Therefore, the extent to which knowledge from the broader teams literature can be applied to fluid teams is relatively unknown. Additional research is needed to understand how fluid teams might differ from more traditional teams in terms of how they function and the factors that contribute to their success. For example, what KSAs should be considered when selecting individuals to be a part of fluid teams? How should individuals be trained to perform within them, and what tools should be utilized? We need to understand which takeaways from the current teams literature can be applied, and what unique recommendations can be offered so that the effectiveness of fluid teams can be better supported.

The purpose of this study is therefore to begin addressing this need by examining fluid team effectiveness within one of the primary contexts in which fluid teams are prevalent—healthcare. Specifically, using a sample of interdisciplinary faculty members and students working in fluid teams within a healthcare facility’s simulation-based education center, we utilize critical incident techniques and thematic analysis to extract themes related to fluid team performance. We then draw from the broader teams literature to identify similarities and differences between fluid teams and traditional teams, and propose recommendations for how the effectiveness of fluid teams can be supported.

## Theoretical background

### Fluid teams

The majority of teams literature addresses more “traditional” teams composed of relatively stable team memberships with members who work interdependently (Bell et al., 2018) and have static long-term memberships (Mathieu et al., 2017), whereas “fluid” teams are more dynamic in nature. Members of fluid teams typically come together on a short-term basis to intensely work together on a single project or task, and then disband once they accomplish their goals (Huckman and Staats, 2011). These members may not have had prior working relationships with each other. Studies have shown that organizations that utilize fluid teams have benefited mostly in fields that are higher-risk and involve a shorter duration of goal attainment, such as in aviation and healthcare (Bushe and Chu, 2011). In the corporate world, organizations that specialize in engineering, professional services, sales, and product development have also reported reaping the benefits of deploying fluid teams.

Limited research does exist informing some of the challenges fluid teams face and various factors that contribute to their effectiveness. In a study of virtual student teams, for example, Dineen (2005) showed that compared to stable teams, fluid teams were more likely to struggle with cohesion and social loafing. Thomas et al. (2018) emphasized the importance of leadership in their model of fluid teams, particularly for generating familiarity and shared mental models, which are otherwise often lacking in these settings. These factors are important for facilitating implicit coordination among team members, and ultimately promoting performance. Though not directly the same as fluid teams, Przybilla et al. (2020) explored membership change within IT teams, and also proposed that team member familiarity and shared cognition would be particularly relevant to the team’s performance. Other factors, such as the centrality of members who are fluid and the timing of the membership change, were also deemed important. Bushe and Chu (2011) highlighted other challenges fluid teams face, including a lack of cohesion and low individual commitment to the group, and proposed several solutions focused on promoting efficacy and belonging in the fluid team context. They also concluded that fluid teams tend to be more effective when there is clear structure in the team and its members have a strong sense of task identity; that is, team members perform the same kind of work and also have clear and established roles, processes, tasks, and goals.

Though not always labeled as such, fluid teams are particularly prevalent within the healthcare context. Emergency medicine, for example, can require teams to quickly come together to perform a complex task, with members often coming in and out based on expertise, emergent needs, and even the shifting availability of medical personnel (e.g., Bedwell et al., 2012). Bedwell et al. (2012) proposed a framework delineating the factors that promote adaptation to membership fluidity within such open medical teams, ultimately facilitating effective team performance. Importantly, this included trainable skills, such as information sharing, shared leadership, implicit coordination, and other more generalizable teamwork skills. In a qualitative analysis of nurses within fluid palliative care teams, Mertens et al. (2019) also emphasized the importance of adaptability. Recently, Akşin et al. (2021) studied fluid teams within the context of ambulance transports. They found that prior exposure among team members was important for performance, especially when the task being performed was less standardized and the workload was higher.

The term “fluid teams” has been used in various ways in the literature, and there are similar terms for teams that share some of their features. We argue that the key distinguishing feature that comes up in discussions of fluid teams is the instability of membership, such that the team does not have a shared history as a team, nor will they have a shared future together as a team, and members can cycle in and out over the course of task completion in response to changing demands. Additionally, although it is possible for some individual members to have prior familiarity or experience working together in other contexts, the team as a whole is largely unfamiliar. Fluid teams are also characterized as being time-sensitive, in that taskwork must begin immediately upon formation and must be completed within a limited timeframe. This is often, but not always accompanied by high-stakes and high stress.

Similar team constructs that have received attention include “*ad-hoc* teams” (White et al., 2018), “swift teams” (Aggarwal, 2014), and “agile teams” (Rietze and Zacher, 2022). Similar to fluid teams, *ad hoc* teams differ from the traditional structure such that team members with no prior experience working together convene to perform a task and then disband (Salas et al., 2008). These tasks are not necessarily high-pressure or time-sensitive, however, thus can include such things as students performing a task in a laboratory setting, or a taskforce coming together to address a current issue in an organization. In the healthcare literature, the term *ad hoc* has also been used to describe teams where membership varies, and certain roles are taken on by different team members over time, such as in academic teaching hospitals (White et al., 2018). Like fluid teams, members lack familiarity and work together for a limited period of time, but their short duration may be due to the nature of shiftwork rather than a time-sensitive task. Further, fluid teams are distinct in that roles are more emergent and dynamic as opposed to having stable positions that are filled by different people at different times.

Also quite similar are, swift teams, or sometimes referred to as “swift starting action teams” (McKinney et al., 2005; Aggarwal, 2014). These teams are composed of highly-skilled, often diverse experts who have no prior experience working together and must perform a high-stakes task immediately following team formation (Wildman et al., 2012). Examples include military combat units, airline crews, and top management strategic decision-making teams, as well as others that extend to the healthcare context, such as trauma response teams. These are more specific than the broader definition of fluid teams in that they are defined by the inclusion of well-trained experts, and unlike fluid teams, dynamic membership post-formation, where members may cycle in and out, is not a key feature.

Although the terms might imply commonality, agile teams are actually quite different from fluid teams. A main feature of agile teams is a work style that revolves around streamlining processes and functioning (Rietze and Zacher, 2022), as opposed to membership dynamism in fluid teams. Specifically, agile teams are characterized by self-organized teamwork, iterative planning, incremental planning and delivery, and retrospective reflection. Finally, the term membership change is not completely independent of fluid teams, as they often have team members coming in and out, but it is often used to describe situations where there is a more stable, ongoing team that loses and gains a member over time (Bedwell, 2019).

In sum, fluid teams share commonalities with other team structures and they are not necessarily mutually exclusive. Fluid teams may encompass some of the features of these other team types, and in practice, it is likely that teams can vary in the extent they are fluid, such that some may work together for a shorter period of time versus others, and some may have more team members cycling in and out in comparison to others. Fluid teams and other similar team formations are increasingly common in the modern workplace, and have begun to receive attention in the literature, but much remains to be learned. In particular, given the structural and contextual differences between fluid teams and traditional teams, it is important to gain a better understanding of how such teams differ in terms of the factors that contribute to their functioning and overall effectiveness.

### Simulation as a context for studying fluid teams

Simulation has been widely adopted in healthcare within the last two decades in order to improve patient safety (Gaba, 2004). Gaba (2004) defines simulation as “…a technique…to replace or amplify real experiences with guided experiences that evoke or replicate substantial aspects of the real world in a fully interactive manner.” Previous research has emphasized the importance of team training in high-stake environments such as Crew Resource Management (CRM) training, which is a widely adopted team training method created to reduce pilot errors in the aviation industry (Helmreich et al., 1999), as well as Team Strategies and Tools to Enhance Performance and Patient Safety (TeamSTEPPS), which was developed by the Department of Defense (DOD) and the Agency for Healthcare Research and Quality (AHRQ) to practice teamwork and improve the quality and safety of patient care (King et al., 2008). For education purposes, simulation is regarded as a valuable tool for developing healthcare professionals’ technical and teamwork competencies in a manner that addresses practical constraints while minimizing risk and other ethical concerns (Lateef, 2010).

Simulation-based education represents a valuable setting for examining fluid teams, as it requires team members with limited familiarity to come together in an *ad hoc* manner, perform tasks that are often time-sensitive or high-stress, and then disband following the completion of the simulated task. In this study, we sampled both students in the healthcare field who participate in simulations as part of their education, as well as faculty members responsible for observing simulations and performing formative assessments of the simulation. These faculty reported many years of experience observing simulation-based fluid teams and providing constructive feedback following each observation through leading debriefs where they discuss what went right and what went wrong during the team’s performance episode. Faculty members are trained healthcare professionals who have also been a part of numerous real-world fluid teams in practice. Based on these credentials, we therefore consider them to be subject matter experts (SME’s) on fluid team effectiveness, at least within the healthcare context.

Considering our focus on healthcare and simulation, which is typically designed to reflect higher-stakes scenarios, this work is applicable specifically to fluid teams that operate under high stress conditions. While all fluid teams produce stressors to some degree due to their defining features (e.g., lack of familiarity), some may work on lower-stakes tasks, such as fluid teams within product development or sales and customer support (Bushe and Chu, 2011). It is also important to note that not all teams within the healthcare context are necessarily fluid. For example, healthcare teams have been classified into four different types, depending on the stability of the roles and personnel that comprise them (Andreatta, 2010). Such teams can have stable roles with variable personnel, variable roles and variable personnel, stable roles and stable personnel, or variable roles, with stable personnel. The latter two would not be considered fluid teams, as the personnel are stable (e.g., private practice, home healthcare teams). Thus, we focus specifically on healthcare teams that are both fluid and high-stress.

## Methods

This study is a descriptive, qualitative thematic analysis of typed (text-centered) reflections submitted by participants of simulation-based education, from the perspective of both students and faculty. The reflections were captured via an electronic survey prepared by the researchers and distributed via a QR code inside the simulation lab and/or a URL link through a commercially available web-based survey software platform (Qualtrics) via email. Participants were told they were free to voluntarily complete the survey and that it would have no impact on the grades of the students or employment of the faculty. Participants consisted of 12 students enrolled in both undergraduate and graduate healthcare programs in the New York Metro area, as well as 24 faculty members responsible for observing their students’ simulation-based education encounters at their organization’s training center. Participants spanned various healthcare professional backgrounds such as Doctors of Medicine (MD), Doctor of Osteopathic Medicine (DO), Advanced Practice Nurses (APRNs, most commonly nurse practitioners), Physician Assistants (PA), Registered Nurses (RN), EMT-Paramedic and residency or fellowship programs.

Utilizing the critical incident technique (Flanagan, 1954), we prompted participants to think about their experiences observing or participating in fluid teams within the context of simulation-based education, and to describe examples of particularly effective and ineffective teams, including the context, the behaviors that occurred, and the consequences of that behavior (survey questions are listed in Appendix). We also asked faculty to report on the KSAO’s they believe are critical to success in this context, as well as their perceptions of the greatest challenges or barriers to effectiveness. We then utilized thematic analysis to assess patterns across survey responses (Braun and Clarke, 2006). Upon data collection, the coding team (composed of 4 members) utilized Microsoft Excel to organize the participants’ responses and sort through their qualitative data. The coding team was composed of two PhD candidates and one PhD with backgrounds in Industrial-Organizational Psychology, as well as one DO with a background in Emergency Medicine and Simulation-Based Education. Coders performed a read-through of all responses and logged internal memos when familiarizing themselves with the dataset before coding began. Following this initial read-through, the first round of coding consisted of open coding in a standardized Excel workbook that was not visible to other members of the coding team. Initial codes were then combined into one Excel workbook to analyze for frequency and followed by interactive discussions among the coders. Frequency counts were then aggregated from each coder to determine unique codes and consistency of codes, rectify discrepancies due to language, and identify outliers. The next round of coding consisted of collating the initial codes into potential themes, defining those themes, identifying relevant subthemes, and determining whether the main theme was related to one of three main categories that emerged: context, behaviors and states, or KSAOs (knowledge, skills, abilities, and other). The coders then came together to establish the final list of themes and reached a consensus on the main themes along with their subthemes.

## Findings

Coding and thematic analysis procedures resulted in the identification of several key themes pertaining to fluid team performance. Our survey questions, which closely followed the critical incident technique, prompted participants to report on the context of, and the behaviors that resulted in effective or ineffective fluid team performance in their experience. Additionally, we asked about the KSAOs that contribute to, or detract from success. In line with these questions, we thus grouped emergent themes into three overarching categories– conditions, team behaviors and emergent states, and KSAOs. In the following sections, we describe the themes we grouped into each of these categories, including how they either facilitate or act as a barrier to effectiveness in this context, as well as how they align with or differ from the current literature on teams research. Findings and results are also summarized in Table 1.

**Table 1:** Key themes related to fluid team effectiveness and comparison to traditional teams.

Themes	Comparison	
	Fluid teams	Traditional teams
*Roles* There is often ambiguity and inconsistency around member roles that detracts from fluid team effectiveness.	Role assignment is dynamic and time-constrained Planning process has to be conducted for every performance episode—cannot rely on shared history Taskwork often begins without clear role assignment Members are likely to have highly specialized skill sets and are less interchangeable—confusion around roles is especially damaging	Typically have more time to devote to team charters or other planning processes Progress through developmental phases that enable them to solidify roles through role compilation process Over time, members develop a clear understanding of role distributions and interdependencies, such as leadership
*Physical stressors* Chaos and physical stressors in the work environment make it challenging for fluid teams to function.	Typically operate in high stress environment, including time–pressure, high-stakes, and complex tasks Impact of stressors can be exacerbated by other features of fluid teams (e.g., lack of familiarity, role ambiguity) Members may experience cognitive load and emotional stress that can impact capacity to perform	Can work in stressful conditions, but less prevalent Typically work within less volatile environments that are more consistent and familiar to members Less likely to experience depletion in cognitive and emotional resources in a time-constrained, high-stakes manner
*Leadership* Success of fluid teams is heavily dependent on the ability of one clear leader to emerge and guide task performance effectively.	Important for there to be one specific leader at a time to reduce confusion and provide clear direction May experience more transitions of leadership due to dynamic nature of fluid teams, but clear transferring of leadership from one person to another is critical—simultaneous sharing of leadership is not beneficial Greater need for task-focused as opposed to person-focused leadership	Leadership in many forms (e.g., internal, external, formal, informal) is important for team effectiveness Shared leadership is particularly beneficial, especially for helping teams be adaptive Person-focused as effective, or sometimes more effective than task-focused leadership
*Communication* Continuous, clear communication and check-in is needed to help fluid teams navigate complex conditions.	Communication is especially critical due to the conditions fluid teams operate within (e.g., more communication is needed since there is not a shared history to draw from) Additional barriers to communication are present, such as lack of leadership, high-stress, and time pressure Explicit, closed-loop communication is necessary Lack of familiarity of team members can result in lack of psychological safety to voice concerns	Communication is a core aspect of teamwork that is necessary for teams to perform effectively There is a shared history that enables teams members to engage in more implicit communication, where there is more anticipation of other members’ needs and adjusting without explicitly communicating about it
*Collaborative processes and states* Members of fluid teams often struggle to develop shared mental models and work together effectively	Often composed of interdisciplinary team members that use different terminology and have different ways of doing things, creating a lack of shared mental models Silos based on interdisciplinary backgrounds can form, reducing collaboration across the whole team Team members are often unaware of each other’s strengths and weaknesses, thus do not know how to work together Training backgrounds focused on individual performance and hierarchical dynamics can result in individuals trying to dominate the team process without collaboration	Collaborative processes and states such as coordination, cooperation, shared cognition, and minimal conflict are important for team effectiveness Work history and familiarity of members may increase awareness of team members’ knowledge and/or specializations, facilitating the ability to collaborate
*Socio-emotional processes and states* Due to the lack of familiarity or shared history, fluid teams can struggle to develop trusting and psychologically safe climates	Requires quick development of trust and comfort with new members who have not previously worked together No shared history to draw from—members may develop trust and psychological safety based on further removed factors, such as personal proclivities, preconceptions, and external sources of information Stressful conditions can create emotionally-charged situations that exacerbate the challenges of developing trust and comfort	Trust and psychological safety are important for performance, learning, creativity, and engagement Teams have shared experiences and established relationships to draw from More opportunity for trust and other affective emotions to grow over time
*Technical knowledge and competence* Members of fluid teams must have the requisite knowledge and skills, as well as an understanding of their role and the roles of other team members to be able to quickly come together and perform.	Lack of shared history produces uncertainty about other members’ competence High-stakes tasks require necessary expertise with less time and margin for errors to allow members to develop their competencies over time Members are often brought in to perform more specialized roles, making it especially important for them to come in with appropriate expertise	Higher familiarity with the roles and specializations that each member is responsible for Members may possess more generalized knowledge and expertise There is time for team members to develop expertise on the job over time
*Readiness and engagement* It is critical for members of fluid teams to be confident in their ability to act while also being receptive to feedback.	It is critical for members to have to the confidence to enter a situation and take action within a limited period of time High-stakes, time–pressure, and uncertainty can result in hesitancy and fear of failure Team members must be receptive to feedback in real time to address changing needs in the dynamic environment	Team climates that emphasize efficacy, potency, and learning goal orientation are beneficial for performance Members have greater familiarity, more time to act, and face lower stakes, making it less likely they will be hesitant to act

### Conditions

Several themes emerged related to the conditions that fluid teams are required to operate within, which primarily involved the importance of roles and the high degree of stressors that are often present within the working environment.

#### Roles

A common theme that emerged in our data was that when fluid teams come together to perform a task, there is often a great degree of ambiguity and inconsistency around members’ roles—common codes included *role designation*, *clear roles*, *unclear roles*, *clear leader*, and *unclear leader*. Many descriptions of ineffective performance and barriers to performance highlighted a lack of clarity on who is doing what, who the leader is, and how each member should contribute to the performance goal. Much of this was due to team members not having any prior familiarity or work history with one another, paired with the fact that there is often not enough time, or at least team members did not take the time, to discuss who would be doing what and who would be taking the lead. In some instances, this would result in multiple team members attempting to lead or otherwise perform the same role, ultimately generating confusion. This outcome typically occurred in combination with some of the other environmental stressors that can be present, as further described below. Additionally, due to the dynamic nature of fluid teams, members sometimes shifted between different roles or tried to take on multiple roles at once. There were also times when a need to re-establish roles arose during the performance episode, which could generate more confusion, if not performed quickly and effectively. For example, these issues were highlighted in the following responses.

*“Observed a team during a "code blue" cardiac arrest event. No clear leader emerged and at times there were conflicting leaders—two people were strongly directing different members of the team with opposing goals … Resources were not used effectively as many team members had no identified role and later stated they didn't know how to be a productive member of the team. Others observed they were in the way or distracting. At multiple times, many team members were fixated a task that normally requires one person leading to other priorities not being addressed in lieu and also disorganization of the task at hand due to "too many cooks" …” [EMT, Faculty 1]*

*“Mock code scenario. Multiple [people] trying to lead team or never being clear who the team leader is. Unclear roles with both role hopping and lack of any person in a role ….” [MD, Faculty 2]*

Conversely, descriptions of effective teamwork often referenced clarity around who was the leader of the team as well as explicit effort to designate roles at the beginning of the performance episode. For example:

*“A clear, well-defined leader with predetermined roles ensured team members remained on task and the case progressed through required steps rapidly with all members of the team aware of the expectations and next steps.” [RN, Faculty 3]*

##### Comparison to traditional teams

Roles are critical in any team, and the notion that each team member has specific roles and responsibilities to carry out has long been a defining characteristic of what constitutes a team (Cannon-Bowers and Bowers, 2011). Role clarification and structuring have been identified as important ways to promote team effectiveness in the broader literature (Salas et al., 2015), suggesting there are similarities between more traditional teams and fluid teams in this aspect. What distinguishes fluid teams is the dynamic and often time-constrained nature of role assignment. For example, traditional teams typically devote time to determining who will do what through team charters (Mathieu and Rapp, 2009) or other planning processes (Marks et al., 2001), and may progress through different team development phases, which can further solidify members’ roles through a role compilation process (Kozlowski et al., 1999). By the last phase of team development, members have a clear understanding of the role distributions and interdependencies across the team, including who they should look to for leadership, enabling them to coordinate and adapt to any changes with relative ease.

As our data indicated, fluid teams, in contrast, are often required to approach each performance episode anew, with limited information about what each person brings to the table, or what roles they are qualified to fill. They may dive into the task without taking the time to plan and designate roles and without clarity about who is taking the lead. Further, roles are dynamic (Tannenbaum et al., 2012)—shifts can occur in who is leading or performing each function while a performance episode is underway, and these changes are not necessarily planned for or explicitly discussed.

We also observed that alongside these role-related challenges in fluid teams is the reality that role differentiation may actually hold greater significance compared to traditional teams, at least in the medical context. For example, while members of a software development team may be somewhat interchangeable in terms of the roles each person is capable of performing and the status and level of expertise they bring to the team, fluid teams in healthcare tend to have highly specialized skillsets and credentials that dictate the specific functions they are qualified to perform, and typically operate within a distinct hierarchy. Thus, the confusion around roles may be especially damaging for fluid team effectiveness.

#### Physical stressors

Perhaps a byproduct of the role-related issues, many respondents mentioned loud noise, people talking over each other, distractions, and general chaos in the working environment as factors that diminished the ability of fluid teams to be effective. Additionally, some described the context as physically cramped and instances when team members bumped into each other or otherwise made physical contact, both intentionally and unintentionally. The conditions were captured in codes such as *chaos, noise* and *crowding,* and present in the following response:

*“… There are too many people involved so there is pushing and shoving … There was a lot of shouting to be heard… As a result, the ambulance crew could not give the patient information, causing repetition of the information.” [EMT/Paramedic, Faculty 2]*

*“Too many people in a room can slow the process.” [MD, Faculty 2]*

##### Comparison to traditional teams

While traditional teams can certainly also work in contexts with a high degree of physical stress, these conditions are much more prevalent in fluid teams, and are exacerbated by other fluid team features, such as the lack of familiarity among members, the role ambiguity and fluidity, and the time–pressure and high-stakes that frequently accompany the task, particularly in the healthcare context. Further, fluid teams in healthcare may spread over a wider geographic location and face additional variability in the environment they are operating in. Our respondents indicated these stressors added to team members’ cognitive load and emotional stress, both of which can detract from their capacity to perform critical team processes effectively. Traditional teams, in contrast, have more consistency and familiarity, making their operating conditions generally less volatile, and less likely to expend cognitive and emotional resources in the same manner.

### Team behaviors and emergent states

The next set of themes we identified and grouped together were related to behaviors and emergent states, or essentially what fluid teams do and the shared states that result, including the actions of individuals and the interactions among the team that contributed to or detracted from team effectiveness. Themes falling into this category included *leadership, communication, collaborative processes and states* and *socioemotional processes and states*.

#### Leadership

Responses indicated that the success of fluid teams is largely dependent on whether one clear leader is present, how successfully this individual takes charge and is recognized by others as being in charge, as well as their effectiveness in delegating roles and otherwise guiding task performance. Common codes that emerged included, *effective leadership, leadership—hierarchy, ineffective leadership, unclear leadership, and coordinator—lack of.* Aligning with the themes that emerged around roles, participants considered it critical to have one clear leader who took accountability and provided direction. Even when it was necessary to have two leaders, a shifting back and forth in which there was distinctly one person acting as the leader at a time, was key. Not surprisingly, it is not enough to just have a leader; this leader needs to be competent. Leadership requires the confidence to emerge, the proper knowledge and skills needed to perform the task, and the higher-level understanding of what is needed and how to effectively assign roles to the appropriate team members to accomplish the goals of the team. Given the ambiguity and high-stress that is often present in the fluid team context, a strong leader appears to be particularly critical for guiding the team through each phase of the performance episode. This was demonstrated in the following example responses:

*“What I observed was all team members who knew of each other, but not the capabilities of the others. Not knowing that, an effective team LEADER needed to be established to effectively organize the efforts of the team to a common goal. Without a dominate hierarchy, the effectiveness of tasks taken are seen and evaluated for their effectiveness. An effective team must designate a team leader to commence the exercise, evaluate the successes and modify the difficulties found for success to be evaluated.” [Paramedic, Faculty 2]*

*“A patient in the emergency department had a seizure. The attending quickly and efficiently called for and then directed different members of the team (pharmacy, residents, nurses …) to do specific jobs.” [MD, Student 1]*

On the other hand, ineffective leadership can contribute further to the team’s struggles, as exemplified in the following:

*“Ineffective leaders with unclear communication lead to incomplete task completion, prolonged time to intervention or time in the trauma bay.” [RN, Faculty 2]*

*“An ineffective team leader can make the communication breakdown. I have seen teams administer two contradictory medications due to an ineffective leader.” [DNP, Faculty 1]*

##### Comparison to traditional teams

In the broader teams literature, leadership has emerged as a major contributor to team effectiveness (Salas et al., 2015). Leadership can entail many different behaviors and can come from individuals both internal and external to the team, and in both formal and informal capacities; but at its core, leadership entails guiding team members toward successful goal accomplishment through whatever means is deemed necessary (Burke et al., 2006; Morgeson et al., 2010). This aligns with what we observed in our sample of fluid teams, in that their effectiveness hinged on the presence of a leader who provides direction to the team, contributes to the task, and takes accountability for the performance of the team.

When more nuanced findings from the literature are considered, however, there are some key differences between fluid and traditional teams. Shared leadership, for instance, when leadership functions are distributed across multiple team members, has been shown to have a positive relationship with team effectiveness, and has even emerged as an approach to helping teams manage complex tasks and respond to changes in situational demands (Aime et al., 2014; Wang et al., 2014). In contrast, our data emphasizes the importance of having one specific leader, suggesting sharing leadership in the traditional way might not be effective in the context of fluid teams. Findings indicate that one clear leader must be present in order to ensure the task is accomplished appropriately. Conversely, respondents considered it a barrier to effectiveness when multiple leaders were present. Although our research also highlighted that transitions of leadership are common in fluid teams, respondents indicated that it was important to have one leader at a time and that smooth transitions were critical. Interestingly, this can potentially be interpreted through the lens of existing work—Lorinkova et al. (2013) found that directive leadership was more beneficial for performance early on in a team’s trajectory, but later on, empowering leadership, when power is shared across team members, became more beneficial. Given the time-constraints and high-stakes often surrounding the performance of fluid teams, there may not be an opportunity for this transition to occur, and more hierarchical forms of leadership may remain optimal throughout.

Another interesting difference that emerged relates to the focus of leadership—research from the broader literature has distinguished between more person-focused leadership, which involves aspects like motivating, empowering, providing individual consideration, and creating a shared vision for followers, and task-focused leadership, which entails providing the structure, resources, and direction necessary for task completion (Burke et al., 2006). Meta-analyses have shown person-focused leadership to be just as, or sometimes even more, important for team effectiveness as is task-focused leadership (Burke et al., 2006). In our data, responses falling into the leadership theme were primarily centered around task-focused behaviors, such as assigning roles, delegating, and even contributing to task performance, with little mention of more person-focused behaviors. We would not go as far as saying that person-focused leadership is not important in fluid teams, but again, given the *ad hoc* and task-critical nature of fluid teams, there may be less capacity for time and resources to be devoted to these functions. Instead, in a time-constrained, often high-stakes situation in which members have no prior experience working together or familiarity with each other’s capabilities, the leader needs to focus on providing the direction needed for the team to function.

#### Communication

Communication, the processes team members use to send and receive information (Salas et al., 2015), emerged as another factor that plays a central role in fluid team effectiveness. Several codes fell into this theme, including *openness of communication,* where there is a bi-directional exchange such that team members freely share information and are receptive to information that is shared with them, *closed-looped communication*, in which team members take specific steps to send information and then confirm that it has been received and interpreted correctly (Salas et al., 2005), and *communication effectiveness*, which captured the quality of communication, or extent to which it enabled the task to be accomplished successfully, and often included a *lack of* communication, indicating poor quality. In addition to the type of communication, responses and corresponding codes also reflected the importance of a continuous back and forth between team members, where they engaged in continuous *check-ins and recaps* during the performance episode to ensure that all members were on the same page, as well as *debriefs* after the performance episode, where there was more emphasis on what could have been done to perform more optimally. These themes encompassed team members both readily offering information, as well as fully *listening* to one other when information is shared. Such findings were present in the following example responses pertaining to fluid team effectiveness:

*“Effective team started off with quick and clear definition of team leader and individual team member roles. Clear and concise closed loop communication and direct communication. Team leader inviting the team to bring forth ideas and concerns (ex. I think this is PEA, does everyone agree[?]).” [MD, Faculty 2]*

*“Interprofessional and multi-disciplinary Trauma team (emergency department, surgery, anesthesia, pharmacy, child life services, xray technician, etc.) assembled at various times during care of a pediatric trauma patient. This was remarkable because the team leader switched from one provider to a second provider during the event. Both team leaders occasionally recapped the scenario, steps taken, and ongoing plan of care. Both team leaders spoke in a calm, concise manner with a normal volume level except when occasionally sternly reminding the team to maintain a quiet atmosphere so communication was easy. They used the call-out strategy where the leader and/or recorder would call-out the next assessment or procedure needed and the appropriate team member would call back the assessment finding or confirmation of the treatment order. The treatments/procedures performed would be confirmed for assignment and completion using the closed-loop strategy. During the debrief, it was clear that the whole team had a shared mental model of the situation, interventions, and outcome of the simulation activity.” [EMT, Faculty 3]*

It was also clear in our data that poor communication was one of the biggest barriers to effectiveness. For example:

*“Lack of interpersonal and communication skills. If you do not know how to speak to people in normal situations, it becomes intensely problematic in higher stress environments. Other team members will immediately turn off to the situation and either consciously or subconsciously undermine the team.” [Paramedic, Faculty 2]*

*“Poor communication is the biggest barrier. Lack of clarity to team lead and roles creates and confusion and lack of direction. Discomfort in bedside staff in speaking up leads to missed opportunities for intervention. Loss of closed loop communication prevents accuracy of interventions or loss of needed intervention.” [MD, Faculty 2]*

##### Comparison to traditional teams

Communication has been defined as “a reciprocal process of team members’ sending and receiving information that forms and re-forms a team’s attitudes, behaviors, and cognitions” (Salas et al., 2015, p. 607), and is considered a significant factor for team success that is very prevalent in traditional teams literature. Our findings on fluid teams align well with broader research, in that communication is considered a core aspect of teamwork that is necessary for the team to perform effectively (Salas et al., 2015).

That said, based on findings, we argue that communication is perhaps even more critical within fluid teams, due to the contextual factors present. Many barriers to communication were identified due to the fluid team environment that resulted in individuals reporting a state of chaos being present and hindering the team’s effectiveness. This was attributed to many factors, including too many team members being present in the room at a time, unclear leadership and a lack of delegation that resulted in excessive talking and noise. Under these conditions, as well as the time–pressure and/or high-stress that is often present in fluid teams, team members may get distracted, miss information, misunderstand each other, etc. More sharing, repeating, and confirming is therefore necessary in comparison to traditional teams in order for fluid teams to be able to function appropriately.

Additional barriers were identified in the codes, including a *failure to listen*, or lack of *psychological safety* to speak up, which will be further discussed below, that negatively impacted the effectiveness of communication in the team. In fluid teams, members are less familiar with each other, which not only results in potential discomfort or uncertainty that may be less prevalent in teams with more consistent membership, but also necessitates more communication than is necessary in traditional teams that have a shared history and existing understanding of what each team member brings to the situation. Interestingly, implicit coordination, when team members are able to anticipate each other’s needs and adjust how they work together without explicitly communicating about it, has emerged as a pivotal process within the traditional teams literature (Salas et al., 2015). Our findings however indicate that in fluid teams, ongoing communication that is explicit and closed-loop in nature is necessary for the team to be able to overcome some of the challenges that are created by their contextual features.

#### Collaborative processes and states

Several additional processes and emergent states came up in our data that reflected the extent the team is on the same page and working together, and were thus grouped under the broader umbrella, *collaborative processes and states*. Responses primarily centered on issues that can occur when members of fluid teams are not working together appropriately (e.g., codes *lack of coordination*, *lack of collaboration*), such as making errors, or the task otherwise not being performed in an effective or efficient manner. This included instances where there was *one person dominating* rather than team members working together. Participants also emphasized the need for the team to have a *shared mental model* up front about the team’s goals, who is doing what, and how the task will be accomplished, and to maintain *shared situational awareness* throughout. Differing ideas about how to do things and other clashes between team members (i.e., *conflict*) were considered barriers to effectiveness, as well as the idea that *silos* based on interdisciplinary backgrounds can emerge in the team that create divides between team members and hinder their ability to work together. These themes are demonstrated in the following example responses.

*“Multiple people were looking after a patient, and would report back to each other and then report findings disparately, rather than seeing or discussing the patient together …” [MD, Student 1]*

*“… when teams fail to communicate, they work in their own silos and it is much more ineffective. The response is disjointed and results in much more back and forth when having to call the provider … patient care suffers. This poor communication results in a much longer time to adequate care. [PharmD, Faculty 1]*

*“Ineffective fluid teams are ones in which different personalities clash and successful operations are ineffective by having all fluid team members working [individually] to showcase their understanding of the team goals and what they individually did vs what the team might not have done as a ‘TEAM’ There is an ‘I’ in TEAM when only one person thinks they are the most important member and must show that off to others. Success is not to be evaluated by 1, but judged by 5 or 6 working for a common goal.” [Paramedic, Faculty 1]*

##### Comparison to traditional teams

The traditional teams literature is ripe with evidence of the importance of things like coordination, cooperation, shared cognition, and minimal conflict for facilitating team effectiveness (e.g., Mathieu et al., 2008; Salas et al., 2015; Mathieu et al., 2019). Our findings on collaborative processes therefore aligned well with what is known about teams in general, but there appear to be some distinguishing features regarding the factors that contribute to, and the likelihood that collaboration issues will occur in fluid teams compared to more traditional teams. For example, fluid teams are often comprised of interdisciplinary team members, which could make it more challenging to work together due to the use of different terminology, norms, ways of doing things, etc. Some particular barriers that fluid teams face related to their collaborative processes is being unaware of the strengths and weaknesses of their less familiar teammates, as well as experiencing silos across different disciplines that are educated and trained separately, resulting in less collaboration. Given the ambiguity and time constraints present, members of fluid teams likely identify with those from similar backgrounds as a way to reduce uncertainty (Tajfel et al., 1979) and focus on interacting with those similar to them to help move forward more quickly than would be possible if they put in the effort necessary to work more collaboratively across all members. Responses also demonstrated an enhanced individual focus present throughout formal education that becomes problematic if carried over into the work setting, and may result in one individual dominating the group or failing to take into consideration other group members.

#### Socio-emotional processes and states

The final theme within the behaviors and states category, socio-emotional processes and states, primarily related to the climate within the team that dictated how team members interacted with one another. For example, many responses described issues around trust, where team members did not feel confident in each other’s expertise or abilities, and were hesitant to accept information from one another due to their lack of familiarity. Themes reflecting a lack of psychological safety also emerged, where there was a discomfort or even fear of speaking up, along with concern about looking bad in front of teammates. This also encompassed the importance of having a respectful climate within the team, and the extent members were approachable and willing to hear from each other. Some of the codes utilized include *psychological safety, trust, respect,* and *comfort*, as illustrated in the following excerpts.

*“The effective teams openly communicate without reservation or fear of being judged. They trust and respect the input from each other. This allows them to work together effectively to come up with a plan moving forward. These plans are much more appropriate with everyone's input compared to one health professional group working independently. The 'correct' answer/response ensues more quickly with that open and trusted communication.” [PharmD, Faculty 1]*

*“When the team doesn’t know each other at all, with inexperienced team members, in an environment where people feel they can’t be honest with each other and feel professionally or personally threatened by each other.” [MD, Faculty 3]*

##### Comparison to traditional teams

These socio-emotional states are also considered core to team functioning across many types of traditional teams (Salas et al., 2015). For example, team trust shows consistent relationships with team performance (Feitosa et al., 2020), and psychological safety also plays an important role in outcomes such as learning, creativity, and engagement (Newman et al., 2017). What appears to be distinct is the development and the role of these factors within fluid teams. On the one hand, trust and similar affective considerations may be more important in ongoing teams than short-term teams – there is more opportunity for trust to grow and for emotions to generally play more of a role over time within longer-term teams, while short-term teams tend to be more task-focused (e.g., De Jong and Elfring, 2010). On the other hand, trust is also considered to be particularly impactful when teams face risk, uncertainty, complexity, and weak structures (Blomqvist and Cook, 2018), as is often the case within fluid teams, because it gives them something to draw from and enables them to quickly work together.

What is more clear, however, is that such states are likely based on different factors in different types of teams. While traditional teams have shared experiences and established relationships to draw from, fluid teams must develop a level of trust and comfort based on other, further removed factors, such as personal proclivities, preconceptions, and external sources of information (Wildman et al., 2012), as well as how the team is doing within the current performance episode (Grossman and Feitosa, 2018). Exacerbating the challenges that come from a having no familiarity or shared history to draw from is the high-stress context fluid teams often operate within—our participants indicated that under the stressful conditions, emotions often ran high, feelings were hurt, and conflicts emerged, making it especially hard to develop trust and psychological safety. The hierarchy particular to the medical context also contributed further to a general sense of discomfort, at least for lower status members.

### KSAOs

The last set of themes that emerged related to certain knowledge, skills, abilities, and other characteristics (KSAOs) members bring to the team that promote effectiveness, or that generate challenges in fluid teams when they are lacking.

#### Technical knowledge and competence

Not surprisingly, many responses indicated the need for each person to have the knowledge, skills, and experience necessary to perform the task in order for the fluid team to be able to function effectively. In addition to this technical competence, they need to understand the correct policies and procedures pertinent to their acting role, as well as the knowledge and skills relevant to other roles in the team. Codes in this category included *requisite knowledge and skills*, *experience*, *understanding of other roles*, and *implementation of policy and procedure*. Responses suggested not only the need for team members to have these KSAOs, but also issues that arise when they are uncertain of the extent other members actually do possess them. These finding were demonstrated in the following responses:

*“… general knowledge of policy & procedure for the unit and task. Understanding of who is involved, what they do and how each role interacts. Knowing where to find needed items (crash cart, IV starts, etc.). Knowing own limits (e.g., ‘I physically can't do adequate CPR, so I will take the recorder role’)…Skills—the required role-specific skills for that task (eg, nursing needs to be able to start an IV, draw blood, do compressions, pass meds; physician needs to be able to interpret labs, lead team)” [CHSOS, Faculty 1]*

*“As much as you hate to admit it, when you add new or inexperienced people into the team it throws off the balance of the team. This is normally witnessed in July when new residents and interns are put into the ERs. They are unfamiliar with how things work, what is required or the ambulance crews that are coming in. It is expected that they will move quickly, quietly and effectively but this is rarely the case. If you're on a Trauma team, its expected that you will have scissors to cut clothing (doesn't happen), its expected that you can jump in and do good CPR (again, doesn't happen).” [EMT/Paramedic, Faculty 3]*

##### Comparisons to traditional teams

Arguably all teams require members to have some level of technical competence for them to be effective. Some barriers identified that are unique to fluid teams included a lack of experience with team members and a resulting uncertainty about other group members’ competence and experience. Compared to traditional teams, individual members of fluid teams are often brought in to perform more specialized roles, and paired with the high-stakes, it is especially important for them to have the necessary expertise. There is also less time or margin for error to allow team members to develop their competence on the job over time.

#### Readiness and engagement

Many responses were indicative of a lack of readiness, in that they conveyed *hesitancy*, *delayed action*, *anxiety*, and even a *complete failure to act* from individuals that detracted from the effectiveness of fluid teams. Conversely, in relation to effective fluid teams, themes emphasized the importance of *confidence*, as well as being *receptive to feedback*, striving for *continuous improvement*, and putting the safety of the patient above one’s own ego, which we interpreted as reflective of a general engagement with the teams’ missions and the broader profession. In order to promote the success of fluid teams, team members must be confident in their ability and willing to take action, as needed. The theme of readiness and engagement was present in a number of codes including *delayed action, confidence* and *hesitancy.* Team members must have self-awareness in order to assess their knowledge and ability, as well as possess a growth mindset to be willing to see potential shortcomings in themselves but proceed to learn, grow and be open to feedback from others. These findings were predominantly present as a barrier contributing to the ineffectiveness of the team and outcomes. Examples of this are included in the following responses:

*“Team members with a lack of confidence to step up into a role for fear of being wrong or failing, even though they knew the right pathway. Led to complete failure of the team objective and frustration felt by all members.” [Paramedic, Faculty 2]*

*“Confidence in abilities, foundational knowledge that supports the team objectives, willingness and acceptance to be wrong, previous experience working collaboratively in teams.” [Paramedic, Faculty 2]*

##### Comparisons to traditional teams

In the broader teams literature, factors such as goal orientation, efficacy, and potency, which align to an extent with the themes we observed, have emerged as important (Collins and Parker, 2010; Chadwick and Raver, 2015). Whereas the focus is often on shared climates and norms within the team, our data highlighted the need for individual members to have confidence and be willing to take action. Further, traditional teams literature may point to social loafing, or the tendency for individuals to exert less effort when working in groups (Simms and Nichols, 2014) as the reason for not stepping up and participating. However, the current study does not appear to demonstrate the same phenomenon. Instead, findings indicate individuals’ lack of performing may originate from uncertainty about how to function in an interdependent role with unfamiliar team members, generating inaction inertia—when the bypassing of an initial action reduces the likelihood that a similar opportunity is taken (Tykocinski et al., 1995). This idea draws from Newton’s First Law of Motion and also encompasses the opposite concept—engaging in action makes continued action more likely.

Given the high-stakes in fluid teams, the avoidance of things that are unpleasant, such as the potential for failure, changes the dynamics of the team and reduces its effectiveness. There is an enhanced focus on loss, resulting in a negative psychological situation where individuals become more hesitant and prone to spare themselves of a potentially negative outcome (Tykocinski et al., 1995). Decision avoidance may also result from a conservation of energy (Anderson, 2003), where members of fluid teams first direct their resources toward trying to understand the situation, who they are working with, what their role is, etc., since they do not have a shared history to draw from. This initial delay may then spiral into further action inertia. Whereas traditional teams work together over time and may have periods of working separately then coming back together, fluid teams are more likely to actively work together throughout the performance episode due to the time constraints and often the nature of the task (e.g., surgical procedure). Members’ delay or failure to act thus may become particularly salient within the team. For this reason, there is also likely more opportunity for individuals to demonstrate how they respond to feedback and try to improve, and more of a need for them to actually use that feedback and adapt in real time, harkening back to the other growth-related themes that emerged within this category.

## Recommendations for practice

While some of the present findings indicate similarities to traditional teams literature, there are considerable distinct features that make fluid teams unique, especially within the study’s context of simulation-based education in the medical field. With this in mind, we present several recommendations based on the specific themes that emerged in our data, and then discuss some broader approaches in the literature related to the improvement of traditional team performance that may be applicable given our results (summarized in Table 2).

**Table 2:** Specific practical recommendations for promoting the effectiveness of fluid teams.

Factors to focus on	Practical recommendations
***Managing the context***	
*Roles*	Develop systems for how teams can establish roles quickly and clearly E.g., training as a transportable skill E.g., procedures and tools Systems should be applied to initial role designation and in response to necessary shifts during performance episode
*Physical stressors*	Adopt high-fidelity training environments that mirror the psychological conditions that will be present in the real work environment
***Promoting key processes and emergent states***	
*Leadership*	Develop systems to help teams quickly identify one clear leader and handle leader transitions E.g., based on shifts or credentials E.g., training focused on explicit communication E.g., tools such as logs, scripts, badges, etc. Encourage hierarchical and task-focused leadership rather than shared or person-focused leadership Train potential leaders on the roles and credentials of other members and the interdependencies across roles
*Communication*	Train and encourage team members to engage in continuous, explicit, closed-loop communication
*Collaborative processes*	Utilize training (e.g., cross-training, frame-of-reference training) to orient team members to the backgrounds, duties, norms, etc. of other members they may work with Anchor members’ identities to a broader mission or purpose rather than allowing them to default to their disciplinary backgrounds or expecting a team-level identity to emerge E.g., build identity through policies and procedures, organizational artifacts, reward structures, etc.
*Socio-emotional processes*	Train and encourage leaders to generate familiarity among team members and set the tone for psychological safety and trust E.g., encourage open discussion, take personal risks Offer metacognitive training to help members become more aware of and in control of their emotions and cognitions Implement initiatives to develop familiarity among people across the organization who might be called upon to work in fluid teams Develop a strong organizational culture that can trickle down into fluid teams
***Selecting for and cultivating KSAOs***	
*Technical knowledge and competence*	Design selection and training procedures to maximize technical competence of individuals who may participate in fluid teams Offer training to inform members of organizational policies and procedures, as well as the technical expertise of other fluid team members Utilize cognitive aids to address differing levels of experience and communicate organizational policies and procedures
*Readiness and engagement*	Encourage a growth mindset in members of fluid teams to reduce hesitancy and encourage receptivity to feedback
***Broader approaches to supporting fluid team effectiveness***	Adopt interprofessional education to reduce collaboration challenges related to team members coming from interdisciplinary backgrounds Utilize simulation-based training to develop experience operating within high-stress settings Offer team training to develop critical collaboration skills such as communication and coordination Incorporate opportunities for social emotional learning (e.g., emotional intelligence training)

### Specific recommendations

#### Managing the context

Given the themes that emerged around roles, it is critical for organizations that utilize fluid teams to develop systems for how team members can establish roles, including who will serve as the leader, quickly and clearly. This could be accomplished through training, where individuals learn how to do this as a transferable skill they can apply to any team they become involved in, as well as through specific procedures and tools that are available to facilitate the process. These mechanisms should apply to role designation at the beginning of the performance episode, as well as during the episode while it is underway in response to any shifting needs that arise along the way.

Regarding the physical stressors in the fluid team environment, we suggest enhancing the training environment to improve fidelity such that it reflects the environment the teams will be operating in to produce similar psychological effects of the stress and workload (Liu et al., 2008). By training in environments that have similar cognitive load and environmental demands, individuals may be better prepared to handle stressful situations moving forward. Furthermore, to reduce the reported “noise” in the environment, we propose several other recommendations that can help, such as those related to role designation and communication.

#### Promoting key processes and emergent states

##### Leadership

Our findings can inform several recommendations for how leadership should be approached and what leadership should entail in order to promote the effectiveness of fluid teams. First, in line with our previous discussions about roles, it is critical for organizations to put systems in place to ensure fluid teams are able to quickly identify one clear leader and to handle leader transitions effectively when needed. For example, members of fluid teams could be trained to know that the first thing they should do upon team formation is to establish who the leader is, and mechanisms should be put in place to inform how they should be making that determination (e.g., credential-based? shift-based?). Team members should also be trained to explicitly communicate and re-establish leadership in situations when shifts become necessary. Beyond training, these processes can be facilitated through different tools and resources (e.g., logs, scripts, badges, etc.) that prompt them, assist them, and hold them accountable for taking certain actions.

It is important to note that our recommendation to adopt a more hierarchical style, where there is one clear leader at a time, may run counter to current thinking in the literature. As discussed, shared leadership is viewed as highly beneficial for traditional teams (e.g., Wang et al., 2014), and has even been argued for in relation to fluid teams—Bedwell et al. (2012) proposed that fluid teams should be trained to distribute and rotate leadership in order to promote their ability to be adaptive. Based on our findings, we would add a caveat to this proposition. To promote adaptability, we suggest that individuals who will work in fluid teams should indeed be trained to enact leadership functions, so that multiple members are capable of performing them. However, once a performance episode begins, they should also be trained to designate one person as the leader at a time and should only shift when a need arises, rather than defaulting to distributing leadership functions throughout.

Regarding what the designated leader should be doing, they need to have the requisite knowledge and skills to perform the task effectively, but as part of their training, they should also develop an understanding of what other team members bring to the table based on the credentials, ranking, etc. within that organization. A deep understanding of the task and associated interdependencies is needed to be able to quickly assign roles and delegate appropriately. Leaders of fluid teams need to have a certain degree of familiarity with each team member and to help team members develop some familiarity with one another (Thomas et al., 2018).

##### Communication

Previous research has suggested practical guidelines to address communication issues, including using closed-loop communication and focusing on sharing unique, rather than common information (Salas et al., 2015). Individuals operating in fluid teams should utilize a closed-loop communication model, which originated through military radio communications, to help ensure that the team understood the message. It consists of three-steps, including communicating the message with a targeted name for its intended receiver, the receiver acknowledging the message, and the individual who provided the original communication ensuring it was accurately received (Salik and Ashurst, 2022). This communication style may reduce the unclear instructions and responsibilities that were reported by respondents.

Research has also focused on information sharing (IS), including both the openness of IS and the uniqueness of IS. According to Mesmer-Magnus and DeChurch (2009), openness of information sharing may not necessarily enhance the knowledge of the team, but it can have indirect effects such as those related to its interpersonal functioning. Conversely, unique information sharing refers to distinct knowledge that a member brings forward for the team’s benefit. Given the lack of familiarly that exists within fluid teams, we propose both open and unique information sharing are vital to the effectiveness of the team.

##### Collaborative processes and states

Based on our findings and existing research, we recommend several pathways to help improve collaborative processes and states in fluid teams. First, current traditional teams literature has emphasized cross-training, where individuals are trained to some degree on the duties of other team members (Volpe et al., 1996). This helps the team develop shared mental models, in turn, facilitating coordination, and ultimately performance (Marks et al., 2002). In a similar vein, research on multi-team systems, has shown that frame-of-reference training can help get subteams with differing specialties on the same page, also promoting cross-team coordination and performance of the broader system (Firth et al., 2015). Thus, some type of training designed to orient team members to the different backgrounds, duties, norms, etc. other members are likely to come in with would be beneficial.

Second, given the tendency for silos to form in fluid teams, paired with the fact that such teams do not have a shared history to draw from and their time together is limited, efforts should be made to anchor their identity to a broader mission or purpose rather than allowing it to simply default to their disciplinary backgrounds. For example, the organization can cultivate a culture where the ultimate goal is to maximize patient outcomes, and every fluid team that forms could then identify with that broader goal and automatically have it in common when they come together. Policies and procedures, organizational artifacts, rewards structures, etc. could all be designed to emphasize the broader mission rather than any single team or individual. On a smaller-scale, but related example, Homan et al. (2008) found that diverse teams perform better when their reward structure emphasizes a superordinate identity rather than subteams based on differences.

Lastly, several of the other recommendations we describe in this paper should help alleviate collaboration issues in fluid teams. For example, a strong team leader can help familiarize team members with each other, create a shared vision for the performance episode, especially by linking it to the broader organizational mission, and encourage participation and interaction from all team members. Effective communication can help get team members on the same page and facilitate coordination. Positive socio-emotional processes (discussed below) can help team members develop more consideration for one another and contribute to a more collaborative atmosphere.

##### Socio-emotional processes

Past research sheds light on specific approaches through which positive socio-emotional processes can potentially be cultivated within fluid teams. Based on their theoretical framework of trust within swift starting action teams, for instance, Wildman et al. (2012) suggested that leaders have an important role to play in helping these teams develop trust. Because they proposed that trust is largely based on emotional reactions and prejudices in these types of teams, leaders can help team members become aware of the source of their biases and guide them toward more accurate judgements of their teammates. Similarly, in a review of literature specifically on healthcare teams, leadership behavior emerged as a key enabler of psychological safety (O’Donovan and Mcauliffe, 2020). Leaders can also help set the tone for psychological safety in the team by encouraging open discussion, taking personal risks, and working to flatten hierarchical differences. Familiarity was another major contributor that emerged, but given the lack of familiarity in fluid teams, leaders can offer further support by helping team members develop a basic level of familiarity, as proposed by prior authors (Thomas et al., 2018). All of this suggests that anyone who might take on a leadership role within fluid teams should be trained on how to enact these specific functions. Providing other types of training to members of fluid teams may also prove beneficial. For instance, metacognitive training could help team members become more aware and in control of their emotions and cognitions that can contribute to socioemotional challenges within fluid teams (e.g., Wildman et al., 2012).

At a broader level, organizations who utilize fluid teams might consider initiatives to help develop familiarity among different people across the organization who might be called upon to work in fluid teams so that even when a fluid team as a whole has no prior history together, specific members might have some prior familiarity with one another that could serve as a foundation for trust. Likewise, if the organization has a strong culture that supports psychological safety, that can be transferred to the individual fluid teams. O’Donovan and Mcauliffe’s (2020) review revealed that when the organization had a culture that prioritized patient safety and encouraged members to speak up about any concerns related to patients, specific healthcare teams within the organization were more likely to experience psychological safety.

#### Selecting for and cultivating KSAOs

To help fluid teams operate more smoothly, organizations’ selection and training procedures should be designed to maximize the technical competence of individuals who may participate in fluid teams. As part of their training, they should become well-informed of organizational policies and procedures, as well as what technical expertise they can expect from their teammates in different roles. If such standards are in place, individuals can come to expect competence even from unfamiliar members, and then become even more confident through efforts to cultivate familiarity from the team leader, as well as through their interactions during the performance episode. Additionally, we propose that cognitive aids may be another pathway to bolstering individual performance and addressing differing levels of experience among the team, particularly in relation to an organization’s policies and procedures. A review of the literature on cognitive aids describes them as prompts designed to assist with task completion, which are created from established guidelines and may be presented as posters, flow charts, checklists or mnemonics (Marshall, 2013). This review yielded evidence that cognitive aids may assist with task completion in the context of medical emergencies in certain situations.

We also suggest team members should adopt a growth mindset to address their potential lack of confidence or certainty in their ability. Dweck (2016) introduced the growth mindset as the belief that intelligence can be developed, the opposite of a fixed mindset where individuals tend to avoid situations that present a threat of failure or struggle that can jeopardize perceptions of their intelligence. Growth mindset beliefs have been a focus in educational settings, where curriculum and structured programs are used to develop it. Research has shown that growth mindsets, or the belief one’s intelligence could be developed, allow individuals to outperform those who believe their intelligence is fixed (Dweck, 2015). While this technique is primarily utilized in education spaces with students, we propose it can be applied in the context of professional development for individuals who participate in fluid teams to encourage continuous learning and reduce the hesitancy and fear of failure that was reported by respondents.

#### Broader approaches

Beyond the specific recommendations we propose, we also suggest there are several broader approaches than can be utilized to support many of the factors deemed pertinent for fluid team success.

##### Interprofessional education

For example, Interprofessional Education (IPE) models should be adopted in education to better prepare individuals to enter the workforce and be members of interdisciplinary teams. Further, the IPE model can be utilized by organizational development teams and training centers. The World Health Organization (2010) (WHO) Framework for Action on Interprofessional Education and Collaborative Practice states: “Interprofessional education occurs when two or more professionals learn about, from and with each other to enable effective collaboration and improve health outcomes.” This approach would arguably create several pathways to help improve collaborative processes in fluid teams—a literature review on the topic of IPE within the context of simulation-based education, referred to as Interprofessional Simulation Education (IPSE), concluded that research is in agreement about its benefits at the undergraduate level, revealing outcomes such as increased confidence, knowledge, leadership, teamwork and communication skills (Gough et al., 2012). The integration of simulation-based education addresses many of the previous findings.

##### Simulation-based training

Simulation-based training may address many of the contextual stressors reported throughout our data by enhancing the training environment to improve fidelity and reflect the environment teams will be operating in to produce similar psychological effects of the stress and workload, among other factors (Liu et al., 2008). By allowing fluid teams to train in environments that have similar cognitive load and environmental demands, individuals may then be better prepared to coordinate and execute in real settings. Cook et al. (2011) conducted a comprehensive synthesis on technology-enhanced simulation, which utilizes various technologies, including computer simulators, high fidelity mannequins, and human cadavers—this approach consistently shows positive relationships with key knowledge, skill, behaviors, and even patient outcomes. At the individual level, simulation-based education supports the acquisition of both technical and non-technical knowledge (Cook et al., 2013, 2018). At the team level, simulation-based training has been reported to improve performance specifically related to communication and other critical teamwork skills (Weaver et al., 2010; Forse et al., 2011). Simulation thus acts as a vessel to improve collaborative processes and develop the KSAs to more effectively manage the stressors presented in fluid teams.

##### Team training

Like traditional teams, we propose that team training can assist in the development of communication and coordination skills that affect the success of fluid teams. Team Strategies and Tools to Enhance Performance and Patient Safety (TeamSTEPPS), for example, is a research-based approach to team training within healthcare that focuses on developing trainable skills including leadership, situational monitoring, mutual support and communication (King et al., 2008). Research has noted that team structure cannot guarantee teams will operate together successfully, and the training may enhance a commitment to shared knowledge to develop KSAs without the need to have permanent assignments (Morey et al., 2002). TeamSTEPPS: Research/Evidence Base (2008) highlights the importance of team training for fluid teams by stating that, “Teamwork does not require that team members work together on a permanent basis, yet it is sustained by a commitment to a shared set of team knowledge, skills, and attitudes (KSAs), rather than permanent assignments that carry over from day to day.”

##### Social emotional learning

Positive socio-emotional processes can help team members develop more consideration for one another and contribute to a more collaborative atmosphere. This recommendation addresses the interprofessional elements of the required KSAs that were found to impact the effectiveness of teams. Social Emotional Learning (SEL) has become an important focus in the nation’s classrooms around the country throughout K-12 education, with enhanced focus on building students’ self-awareness, self-management, social awareness, relationship skills and responsible decision-making (Abrams, 2023). While SEL has been controversial in K-12 education, we propose that individuals working within fluid team environments may benefit from these topics to address the difficulty navigating the interpersonal functioning within a team. Cherry et al. (2014), for instance, propose the importance of emotional intelligence in medical education, explaining that, “Emotional intelligence (EI) is a term used to describe people’s awareness of, and ability to respond to, emotions in themselves and other people.” EI can help individuals cope with the demands and stress within their environment and is composed of non-cognitive competencies including perceiving emotions, using emotions, understanding emotions, and managing emotions (Johnson, 2015). EI is noted to be an ability-based skill that can be developed through targeted training and has the potential to positively impact many relationships within medical education, including between colleagues operating in fluid teams.

## Additional discussion

This study makes several important contributions to both research and practice. First, we identify a set of key factors that can promote or detract from the effectiveness of fluid teams. While some research on fluid teams has been accumulating and certain variables and relationships have been examined, this work advances knowledge by taking a higher level approach to understanding fluid team effectiveness overall. Our qualitative exploratory approach was fitting for generating new understanding (Edmondson and Mcmanus, 2007), such that we allowed themes to emerge from the data rather than coding based on an existing framework. As the use of fluid teams continues to grow in the modern workplace, it is critical to understand how they function and perform—this research helps address the current gaps in our knowledge.

Although we used an emergent approach, it is encouraging that our results do align to an extent with existing research on teams in the healthcare context. Gregory and colleagues (2019) for instance, conducted a review of existing frameworks for healthcare teams and extracted several features they argue characterize “perfect medical teams,” in terms of their ability to be adaptive. Several themes similar to ours emerged, such as the importance of establishing an awareness of roles, fostering psychological safety, and enacting effective leadership behavior, among others. Our research lends further support to many of their ideas and expands on it by focusing on fluid teams specifically, and making comparisons to traditional teams to support deeper understanding. In doing so, we identified unique themes as well, such as the critical need for members of fluid teams to possess certain characteristics, including technical competence and readiness. Although it may seem like a given that teams should be composed of members who are able and willing to perform the task, our results highlighted how intricate knowledge of the task, including other members’ roles and the interdependencies between roles, is needed to help the team quickly act and trust one another without having prior familiarity. Confidence was also key for helping members take action in high-stress, ambiguous situations. Thus, our insights both converge with, and expand upon current knowledge on teams in the broader healthcare context.

Second, we compared our findings to those from the broader teams literature, enabling us to provide insight about the extent to which existing knowledge can be applied to fluid teams, including that which informs how they can be supported. Our results reveal that several major takeaways from research on traditional teams can indeed be applied. For example, factors like leadership, communication, and shared mental models that have been deemed core to team functioning in the broader literature emerged as critical for fluid teams as well. However, these factors often showed up in nuanced ways, and numerous differences between fluid and traditional teams were also present. Thus, we identify several specific areas where we should not just assume that existing findings can be applied to fluid teams as is, but rather, where additional consideration is needed. These findings can inform future research needed in this area, such as the development of a theoretical model of fluid team effectiveness, and empirical studies designed to expand our understanding of the specific factors deemed critical to success, and more precisely test the differences between fluid and traditional teams.

Lastly, we draw from our findings to present a set of recommendations for how the effectiveness of fluid teams can be supported in practice. These span specific approaches that can be used to target certain elements of effectiveness, as well as broader approaches that help bolster fluid team success more broadly. These recommendations can serve as both a valuable resource to organizations utilizing fluid teams, as well as a foundation for future research in which the efficacy of each approach is more directly examined empirically. Such efforts can also help identity any gaps or barriers to providing solutions to address fluid team effectiveness.

There are of course limitations of this research to consider, including the smaller sample and the approach of assessing fluid teams within a training environment, where there may be fewer and different contextual factors present than in the natural setting. We recognize that simulation-based education may filter some of the fidelity that fluid teams experience, specifically in relation to the lack of interdisciplinary training, as students participating in this study were part of homogenous fluid teams as a component of their formal education. In this way, it is possible that the contextual factors related to the effectiveness of the team were limited; however, faculty respondents may have provided examples of fluid teams in their career experience, outside of the survey questions posed about the simulation training environment. Future research examining fluid teams in a higher fidelity setting as opposed to the simulation-based education environment would help develop our understanding of fluid team effectiveness even further. As mentioned earlier, our findings apply specifically to fluid teams that perform high-stress tasks, and may not generalize to those tackling less extreme needs (e.g., engineering teams).

In a similar vein, the context and population of the data collected may have heightened the appearance of certain themes in the data. For example, there may have been less of an emphasis on requisite knowledge and skills if data were not collected in relation to less experienced students in an educational setting. Responses from faculty who have broader experience with fluid teams, including in medical practice, may have helped balance out this shortcoming. Related to this, we considered whether our findings pertaining to role and leadership ambiguity are likely to generalize, or if they are perhaps an artifact of the simulation-based education context. That is, is it possible that roles are less ambiguous in real-world fluid teams where there might be greater hierarchy and more cross-disciplinary members? After consulting with the SME on our research team as well as the academic literature, we argue that the issues related to role and leadership ambiguity are likely to be just as, or even more prominent in real-world fluid teams in healthcare, for a number of reasons. First, there is a long history of attributing medical errors to teamwork issues, including lack of leadership and poor communication (Kohn et al., 1999), suggesting such concerns are indeed prevalent in practice. Issues of unclear leadership and role ambiguity have also been documented in other high-stakes fluid teams outside of simulation, such as in aviation crews (e.g., Gregorich et al., 1990). Second, simulations are purposefully designed to mirror the way things work in the real-world, and thus in this context are likely to invoke challenges that are similar to those encountered in actual fluid teams (Martin et al., 2020). Simulation has been proposed specifically as an approach to addressing some of the common pitfalls that are encountered in healthcare teams, including a lack of understanding of roles and responsibilities (Lateef, 2010). Third, and related to the prior point, although the simulation-based educational environment used in this study is considered high-fidelity, one aspect that is a departure from the real-world is that it is a controlled environment where teams are operating independent of the broader system. Simulations are typically designed to address very specific learning objectives which creates less ambiguity about the task required of the team, and in turn, the roles needed to perform the task. Thus in reality, uncontrolled conditions would add additional uncertainty about the task and the roles of teammates as well as additional cognitive load, which could exacerbate these issues even further, suggesting that the themes we observed related to leadership and role ambiguity are likely to apply, and perhaps are even more prominent in fluid healthcare teams in practice. That said, we do acknowledge a limitation of our sample was that there was less variance in team members’ experience and disciplinary backgrounds than what typically characterizes a fluid team in healthcare. Studying fluid teams in hospital or other medical settings in future research would provide further insights about how leadership and role ambiguity concerns take shape and are managed.

It is also possible that the findings related to physical stressors are an artifact of the simulation context, but similar themes have been observed in healthcare teams in practice, suggesting findings may generalize beyond simulation. Overcrowding, for instance, has been identified as a factor that contributes to injuries in nurses (Triolo, 1989). Likewise, Rashid and Zimring (2008) conducted a review of environmental conditions that can produce stress in healthcare workers, and several factors were similar to the themes we observed, including noise and the configuration of the room they worked within. Physical stressors such as noise, high altitudes, and extreme temperatures are also likely prevalent in fluid teams that operate within other contexts such as aviation and military settings (Flood and Keegan, 2022; Masi et al., 2023). However, it is important to note that findings related to physical stressors will likely not generalize to all types of fluid teams, such as those in engineering or product development teams.

We also recognize this study seeks to understand fluid team effectiveness without linking the team processes and states to any performance metrics. Although a majority of the sample consisted of experienced educators in simulation-based education, the experiences of effectiveness are ultimately subjective for the purpose of this study. Qualitative findings could be better supported with more objective metrics in future efforts. Lastly, we examined fluid teams within healthcare, but acknowledge that findings may not directly apply to fluid teams in other contexts. Future research expanding this work to other settings would be valuable for increasing generalizability of the findings.

## Conclusion

This research identified specific conditions, behaviors and states, and KSAOs that contribute to the effectiveness of fluid teams, especially in the healthcare setting, and provided insight about how these factors compare to existing literature on traditional teams. As organizations continue to evolve in the ever-changing world of work, it is important for both researchers and practitioners to understand how fluid teams function in order to develop and deploy appropriate solutions to modern-day issues. While there are many similarities in the existing literature on traditional teams, there were notable differences found in the factors that either facilitate or act as a barrier to fluid team effectiveness. Moving forward, we hope future researchers will continue to explore and expand on them, as well as our proposed recommendations for practice.

## Data availability statement

The raw data supporting the conclusions of this article will be made available by the authors, without undue reservation.

## Ethics statement

The studies involving humans were approved by Hofstra University Institutional Review Board. The studies were conducted in accordance with the local legislation and institutional requirements. The participants provided their written informed consent to participate in this study.

## Author contributions

RG: Conceptualization, Formal analysis, Methodology, Project administration, Supervision, Writing - original draft, Writing – review and editing. BB: Conceptualization, Formal analysis, Investigation, Methodology, Project administration, Writing – original draft. JH: Conceptualization, Formal analysis, Writing – original draft. MC: Formal analysis, Writing – review and editing.

## Appendix

Survey questions

Faculty questions

Please think about your experience observing fluid healthcare teams within the context of simulation-based education.

1. Think about a fluid team that you observed that was particularly effective. Please describe the context, the specific behavior(s) that occurred, and the consequences of the behavior.
2. Now think about a fluid team that you observed that was particularly ineffective. Please describe the context, the specific behavior(s) that occurred, and the consequences of the behavior.
3. Across the many fluid teams you have observed, do you think there is any knowledge, skills, abilities, or attitudes that are particularly important for success? Please explain.
4. Across the many fluid teams you have observed, what do you perceive to be the biggest challenges or barriers to effective teamwork in this setting? Please explain.

Student questions

Please think about your experience participating in healthcare teams within the context of simulation-based education.

1. Think about a fluid team that you participated in that was particularly effective. Please describe the context, the specific behavior(s) that occurred, and the consequences of the behavior.
2. Now think about a fluid team that you participated in that was particularly ineffective. Please describe the context, the specific behavior(s) that occurred, and the consequences of the behavior.
